# The bereavement experiences of families of potential organ donors: a qualitative longitudinal case study illuminating opportunities for family care

**DOI:** 10.1080/17482631.2022.2149100

**Published:** 2022-12-05

**Authors:** Sean G. Dicks, Holly L. Northam, Frank M.P. van Haren, Douglas P. Boer

**Affiliations:** a Department of Psychology, University of Canberra, Canberra, Australia; b Department of Nursing and Midwifery, University of Canberra, Canberra, Australia; c Medical School, Australian National University, Canberra, Australia

**Keywords:** “Patient and public involvement”, bereavement, family bereavement, organ donation, posttraumatic growth, family resilience, ICU, DCDD, end-of-life-care

## Abstract

**Objectives:**

To illuminate opportunities for care in the context of deceased organ donation by exploring pre-existing family and healthcare professional characteristics, in-hospital experiences, and ongoing adjustment through the lenses of grief theory, systems theory, meaning-making, narrative, and organ donation literature.

**Method:**

Qualitative longitudinal case studies explored individual and family change in five Australian families who had consented to Donation after Circulatory Determination of Death at a single centre. Participants attended semi-structured interviews at four, eight, and twelve months after the death.

**Findings:**

Family values, pre-existing relationships, and in-hospital experiences influenced first responses to their changed lives, understanding of the patient’s death, and ongoing family adjustment. Novel behaviour that was conguent with family values was required at the hospital, especially if the patient had previously played a key role in family decision-making. This behaviour and emerging interactional patterns were drawn into family life over the first year of their bereavement.

**Recommendations:**

Training that includes lenses introduced in this study will enable healthcare professionals to confidently respond to individual and family psychosocial needs.

**Conclusion:**

The lenses of grief theory and systems thinking highlight opportunities for care tailored to the unique in-hospital context and needs that emerge in the months that follow.

## Introduction

If a seriously injured patient reaches the hospital, their family’s experiences will include interaction with healthcare professionals (HCPs) in an unfamiliar environment (Imanipour et al., 2019; Kentish-Barnes et al., 2015), and if doctors find that treatment would be futile, organ donation (OD) may be raised (Ahmadian et al., 2019a, 2019b; Da Silva Knihs et al., 2020). Although time at the hospital is stressful, opportunities for family members to gather, support each other, and be with the patient (Downar et al., 2020; Fernandes et al., 2015; Kentish-Barnes et al., 2019b; Sarti et al., 2018), while receiving support and guidance from staff, can foster later adjustment (Wiegand, 2012; Wijngaards-de Meij et al., 2008).

While confident decisions to decline or authorize OD do not hinder coping (Ashkenazi & Guttman, 2016; Downar, 2018; Kentish-Barnes et al., 2018), ambivalence or negative in-hospital experiences can lead to anxiety, post-traumatic stress, or complicated grief (Rogier, 2017; Yang et al., 2018). HCPs must therefore facilitate family understanding, treat the patient with dignity, and contribute to a supportive environment (Mills & Koulouglioti, 2016). Support should be available for staff too, as they may experience ethical dilemmas, vicarious trauma, and grief following patient death (Meller et al., 2019).

With research in this context focussing on enabling informed decisions and increasing donation rates (Ashkenazi & Cohen, 2015; Marck et al., 2016), connections between in-hospital experiences and ongoing adjustment are under-explored, especially in relation to Donation after Circulatory Determination of Death (DCDD, Dimo, 2019; Naef et al., 2020; Sque et al., 2018; Takaoka et al., 2020; Zheng et al., 2020). Identifying and responding to family and staff needs in this complex environment are challenging tasks for HCPs (Kerstis & Widarsson, 2020; Shariff et al., 2017; Simonsson et al., 2020; Walker & Sque, 2019; Zellweger et al., 2017), highlighting the need for training to improve end-of-life care and peer support (Chan et al., 2020; Crawshaw et al., 2019; Witjes et al., 2017, 2019a, 2019b).

The current study, developed in the context of a PhD, aimed to identify links between pre-existing family and HCP factors, an unexpected death, in-hospital experiences, and later adjustment to highlight novel opportunities for family and staff care. The suitability of viewing experiences in terms of grief theory, systems theory, meaning-making, and a narrative perspective, in addition to the OD decision, was also explored.

**Table:** 

**What is known?** Compassionate care and clear information that assists families to understand death in this context, the option of organ donation, and organ donation processes, contribute to informed and enduring organ donation decisions. Families appreciate time to consider relevant factors when making their decision.
**What does this study highlight?** Families are at the hospital primarily because of the death or imminent death of a relative. In addition to the above-mentioned forms of support, this unique context provides opportunities and time that could assist them to respond to the death and its implications in ways that foster individual coping, family togetherness, anticipatory mourning, and a peaceful death.
**Why is this important?** The patient can no longer actively participate in family processes. We found that, especially when the family’s usual decision-making patterns were disrupted by the death, novel interaction that was first trialled at the hospital was drawn into family life later if it was useful and fitted with family values. The family’s time at the hospital thereby had lasting implications, regardless of the outcomes of their organ donation decision. We also found that other features of the in-hospital processes and general OD context were drawn into family meaning-making and narrative restoration.
**What are the implications for practice?** Training must increase healthcare professionals’ awareness of grief-related features of the family experience, and foster appreciation of systemic change occurring as the family system adapts. This can increase staff confidence and ability to foster a supportive environment that assists with anticipatory mourning, meaning-making, narrative development, and interpersonal adjustment.

### Relevance of the study

From the HCP point of view, when a patient receiving artificial cardio-respiratory support cannot be saved, and end-of-life discussions with their family are being planned, they are identified as a potential organ donor. One of two pathways to OD could be considered. The family decision about donation after neurologically determined death (DNDD) is made after brain death has been declared, while Donation after circulatory-determined death (DCDD) is considered when, while the patient is not brain dead, injuries make treatment futile. After a potential donor has been identified, further investigation may nevertheless show that there are medical or other factors that prevent OD. Some families will raise the potential for OD based on their own understanding of requirements and processes.

The Australian Organ and Tissue Authority (AOTA) reports that about 2% of patients dying in hospital can donate organs. One donor can save the lives of up to seven patients on transplant waiting lists and enhance the lives of several others through eye and tissue donation (Australian Organ and Tissue Authority (AOTA), 2022). To provide context, Table I shows the number of DNDD and DCDD donors in six developed countries during 2021 and compares rates of donors per million population (DPMP, Global Observatory on Donation and Transplantation (GODT), 2022).

**Table I.** Deceased organ donation in six developed countries during 2021.

Country	DNDD donors	DCDD donors	Total donors	DPMP
USA	9674	4190	13,863	41.64
Spain	1243	622	1905	40.79
France	1392	222	1614	24.68
UK	786	564	1350	19.79
Canada	502	232	734	19.27
Australia	297	124	421	16.32

The number of families approached in each country would have been greater than the number of donors. For example, in Australia (Australian Organ and Tissue Authority (AOTA), 2022), 1250 patients were identified as potential organ donors in 2021, and 1170 families were approached with a request to consider OD (94%). Of the families approached, 655 (56%) consented to OD and in 421 of these cases OD proceeded (64% of consented cases). Each family approached would have included multiple family members, their friends, and other support persons.

Improved understanding of the psychosocial features of this context can enhance practice and care delivery, influencing ongoing adjustment in this unique group. We therefore aimed to identify opportunities for individual and family care at the hospital and thereafter.

## Research preparation

### Guiding frameworks

When considering OD, families consider the *Past*, including family relationships and knowledge about OD; *Present*, (time at the hospital); and *Future*, including implications of decisions (Walker et al., 2013). To investigate coping over time, the current study explored links between pre-existing factors, a critical incident, time at the hospital, and ongoing adjustment. Theoretical frameworks used to make sense of family experiences included grief (Gillies & Neimeyer, 2006; Stroebe & Schut, 1999, 2015; J. W. Nadeau, 2008; Walter, 1996; Worden, 2018), systems thinking (Grisogono, 2006; Mehta et al., 2009; J. Nadeau, 2001), meaning-making (Park, 2010), narrative development (Neimeyer et al., 2010, 2014), posttraumatic growth (Tedeschi & Calhoun, 2004), and family resilience (Walsh, 2002).

### The PhD context

The candidate conducted an initial literature review exploring in-hospital experiences of families of potential organ donors and proposed a model of interaction that described how relationships between family members and staff facilitate features of the in-hospital psycho-social environment (Dicks et al., 2017b). A second review explored the suitability of using the above-mentioned guiding frameworks when researching family bereavement and adjustment in this context (Dicks et al., 2020a). Given that (Dicks et al., 2017b) had highlighted the important role that staff play in family experiences, a third review focussed on experiences of members of the multi-disciplinary team (Dicks et al., 2020a), demonstrating that the in-hospital context is stressful for staff too. It was proposed that training that empowered staff to guide families would build staff confidence, foster constructive interaction between HCPs from different disciplines, and assist them to find meaning in the process independent of the family’s decision about OD. Another review linked literature from both the OD and transplantation fields to explore the connection that develops between recipients and consenting families (Dicks et al., 2018a). In addition, exploration of research addressing psychological issues in this field (Dicks et al., 2018b) facilitated the identification of research priorities, the creation of a research plan, and negotiation with Human Research Ethics Committees (HRECs, Dicks et al., 2020b).

### Working hypotheses

Guiding frameworks and literature reviews contributed to emerging working hypotheses including: Pre-existing factors influence responses to death (Walker et al., 2013); The critical incident and complex IHPs influence ongoing adjustment (Ashkenazi & Cohen, 2015); When death disrupts the family narrative, meaning-making assists the family to make sense of experiences (Park, 2010); Openness and support reduce risk of overload (Stroebe & Schut, 2016); and Where transplants have occurred, the narratives of the donor, their family, and recipients overlap (Galasinski & Sque, 2016).

### Research questions

The overarching research question was, “What are the bereavement experiences of families of potential organ donors?” Supporting questions included: How do pre-existing factors, a critical incident, and in-hospital processes (IHPs) influence bereavement?; How does staff confidence and knowledge influence interaction with families?; How do individuals and family systems adjust to the death and create new paths?; and, for consenting families, What role do thoughts about transplant recipients play?

### Stakeholder and participant involvement

The research team’s understanding was influenced by the first author’s role as clinical psychologist (23 years) and family support coordinator at an OD agency (10 years), as well as his PhD supervisors’ (co-authors) past and present roles as Professor in Psychology, Intensive Care Unit (ICU) specialist, Donation Specialist Nursing Coordinator (DSNC), and nurse educator. Accepting that their prior knowledge and assumptions could influence the research project, the team sought to ensure that the study was relevant to HCPs and families of potential organ donors, and that the final report reflected participant experiences in a trustworthy manner (Bartlett & Vavrus, 2017; Stake, 1995). Demonstrating reflexivity (Begoray & Banister, 2010), boxes throughout the current report show the first author’s monitoring of his influence on research processes, participant experiences, data collected, and analysis.

Research-related activities became part of participants’ experience of the first year without their relative (Dyregrov, 2004; Harrison et al., 2017; Simons, 2009). When they responded to questions, hoping that their stories would help others, and when the candidate acknowledged their responses, he and participants co-constructed the data collected (Brennan & Letherby, 2017; Burles, 2017). Viewing research as one of the family’s experiences, and data co-construction as unavoidable, it was imperative to involve stakeholders and participants during study design, analysis, and reporting (Begoray & Banister, 2010).

Before submitting the research plan for review, input was obtained from families who had previously consented to OD, ICU nurses, DSNCs, and a consumer advocate. Input from families supported a focus on bereavement, and some trialled the proposed data collection format. HCPs confirmed in-hospital complexities and suggested recruitment procedures. The consumer advocate gave guidance about the need for care and respect during recruitment and data collection. After obtaining approval from the Australian Capital Territory Health Directorate and University of Canberra HRECs (ETH.11.16.234), the candidate asked these stakeholders to comment on the approved research plan. The HRECs accepted revisions made in response to stakeholder feedback (Dicks et al., 2020b). During data collection and analysis, participants verified researcher understanding, commented on the thematic coding and suitability of exemplars, and have confirmed that this report reflects their experiences.

**Table:** 

Stakeholder and participant input provided access to suggestions and insights from people with lived experience. This ensured the study’s relevance and highlighted the need for a sensitive approach to data collection. Participant involvement during data analysis and reporting enhanced confirmability and trustworthiness of the research outcomes.

## Method

A qualitative study enabled exploration of lived experiences of families who had made in-hospital decisions about OD (Carolan et al., 2016; Girones et al., 2018, 2015; Stake, 2006; Yin, 2018). The longitudinal Comparative Case Study approach highlighted change over time at different system levels, such as individuals and dyads (Bartlett & Vavrus, 2017), and assisted us to explore processes by which family bereavement and ongoing adjustment unfolded. Cases were defined in terms of the exploration of individual and family adjustment over the first year following an in-hospital death where OD was considered. Data collected at three points during the year enabled comparisons of individual and family functioning before the critical incident and at the hospital (times before meeting the researcher), and over the year that followed, where we were able to observe adjustment directly.

### Inclusion and exclusion criteria

Our definition of “family” included members of the deceased’s immediate family, extended family, and significant others (e.g., friend, romantic partner). In each specific family, those who participated determined the boundaries of the definition. Families who had in-hospital conversations about OD with HCPs were eligible to participate, regardless of who initiated the discussion, or whether OD occurred. Family members under 18 years and adults unable to give informed consent were excluded to reduce risk. To avoid dual roles, families receiving support from the candidate in his family support role at the OD agency were excluded too.

### Recruitment

Recruitment occurred at a single centre in Australia. After considering alternatives and the sensitive environment in the ICU (Kentish-Barnes, 2019a), DSNCs (Potter et al., 2021; Tocher et al., 2019) were chosen to provide families with Participant Information Forms (PIFs) after finalizing discussions related to OD. PIFs described the study and explained that families could initiate or decline participation early or wait to be contacted after three months. Following guidance from the HRECs, it was acknowledged that suicide-bereaved families could be experiencing additional stressors and they were therefore explicitly advised that they could decline the PIF.

### Data collection

Semi-structured interviews were conducted at about four, eight, and twelve months following the death. Interviews were audio-recorded with permission and transcribed before analysis. After introductions, conversations commenced with requests such as “Please tell me about when [Name] was in hospital.” Questions such as, “What contributed to that?” prompted elaboration. At the first interview, genograms (McGoldrick, 2016) were used to explore historical functioning before discussing the critical incident and IHPs. Subsequent interviews explored ongoing adjustment. Our intention was to interview multiple family members simultaneously, enabling observation of interaction in addition to collection of verbal data.

### Family care during data collection

HRECs expressed concern about conducting interviews during the first year of bereavement and highlighted the need to monitor participants’ coping. HRECs also stressed the need to avoid dual roles as the interviewer was a support coordinator at the hospital where families were recruited. To monitor coping and the impact of interviews, but avoid discussions resembling counselling sessions, participants completed standardized instruments following interviews. These instruments did not contribute to quantitative research data.

In this regard, The Depression Anxiety and Stress Scale, a 21-item measure of depression, anxiety, and stress (Antony et al., 1998) and The Bereaved Parent Needs Assessment (BPNA, Meert et al., 2012), a 68-item questionnaire assessing needs following an in-hospital death, were completed after the first interview (permission had been obtained to replace the word *child* with *relative)*. After the second interview, participants completed the Hogan Grief Reaction Checklist, a 61-item instrument measuring the multi-dimensional nature of bereavement (Hogan et al., 2001) and the Grief and Meaning Reconstruction Inventory, a 29-item measure of meaning-made (Gillies et al., 2015).

### Avoiding dual roles

In his family support role, the first author is not involved during the IHPs, and first contacts families in the weeks thereafter. Coping is explored, and support is then tailored to fit family needs. He attends to the forwarding of anonymous correspondence between donor families and recipients when received and obtains de-identified information from transplant centres when families ask about recipient progress. To avoid dual roles, when participants required assistance with above-mentioned tasks, they were assisted by DSNCs, and alternative sources of psychosocial support were brought to the family’s attention in the PIF.

### Recruitment outcomes

Although recruitment had initially been planned for 12 months, when only five families had joined the study, this period was extended. After 16 months, however, recruiting was ceased to enable the candidate to attend to interviews with recruited families, transcribing, analysis, and reporting. During the recruitment period 55 patients at the centre were identified by family or the treating team as potential organ donors and discussions were held regarding OD, making their families eligible to participate in the study. Table II summarizes aspects of the recruitment process.

**Table II.** The potential for family participation and the introduction of the study.

Characteristics of case	PIFs provided	No PIF provided
No DSNC	0	3
Medical factors identified early excluded OD	0	14
Family declined OD	5	12
Medical or logistical factors prevented OD after consent	2	2
OD and transplantation occurred	8	9
TOTALS	15	40

In three cases exploration of OD ceased after discussions between family members and the treating team. The other 52 families were approached by a DSNC (94.5%). In 14 of these cases early assessment found that patients were not medically suitable for OD, and DSNCs felt that it was not appropriate to provide a PIF given that the OD agency would not be having further supportive contact with the family.

When interacting with the remaining 38 families where OD was actively explored, DSNCs did not introduce the study where intra-familial stressors or grief reactions were significant, and the family appeared too vulnerable. One of the families who had received a PIF earlier during the recruitment period experienced a second death that contributed to consent for OD. The family indicated that they were aware of the study and were therefore not given a second PIF. At the end of the recruitment period, 15 families had received the PIF which introduced the study.

After considering OD, 21 of the 38 families consented (55%), and 10 of these families received a PIF. These included two of the four families where, after consent, OD did not proceed for medical or logistical reasons. In three of the eight cases where OD occurred and families received a PIF, OD had followed the DNDD pathway, while in five cases the DCDD pathway had been followed. Among the 17 families who declined OD, 5 received a PIF.

Given that PIFs were not given to families where OD was excluded early, when grief reactions were intense, or where family dynamics contributed to significant complexity and distress, the families who were introduced to the study were those who had been seen to be able to cope with the additional tasks of considering participation, and possibly participating in a longitudinal study at a difficult time in their lives. Diversity within the population of families who could join the study was thereby reduced compared to what had been planned.

Two of the 15 families who received PIFs accessed support from the first author in his family support role, making them ineligible to participate in the study. One family initiated participation shortly after the death of their relative. When the remaining twelve families were contacted at three months, family members from four decided to participate. All five participating families had consented to DCDD and had attended the withdrawal of cardio-respiratory support (WCRS). In one case, OD had not proceeded for medical reasons.

### Participation in the study

Table III demonstrates the level of participation in families and the timing of data collection. In four families, there were participants who completed all stages. In Family D, four members completed stage 1 and planned to continue to stage 2. However, when one of these participants died suddenly, also becoming an organ donor, two family members withdrew. The remaining family member completed all stages. In Family E, the single participant withdrew after stage 1. Those who withdrew had found participation emotionally challenging and were given information about independent support.

**Table III.** Participating families and data collection interviews.

Family	Interview details	Stage 1	Stage 2	Stage 3
A				
	Participants	5	5	5
	Timing	4 months	8 months	12 months
	Type	1 individual in-person 2 individual phone 1 couple on phone	1 individual in-person 2 individual phone 1 couple on phone	2 in-person interviews, each with 3 participants^1^
B				
	Participants	2	2	2
	Timing	2 months	7 months	13 months
	Type	1 in-person 1 phone	1 in-person 1 phone	1 in-person 1 phone
C				
	Participants	1	1	1
	Timing	5 months	9 months	13 months
	Type	in-person	phone	phone
D				
	Participants	4	1	1
	Timing	3 months	7 months	14 months
	Type	2 with 2 in-person	phone	phone
E				
	Participants	1	0	0
	Timing	3 months		
	Type	in-person		

^1^The donor’s widow attended both final interviews.

Considering participant availability and capacity, three interviews (each 60 minutes) were conducted with those participants who completed all stages rather than four as had been planned. Participants lived in different cities, making family meetings impossible. Timing of interviews differed between families but was generally consistent within families. For example, all stage 1 interviews with members from Family A were held during the fourth month after their relative’s death. Most interviews were with individuals or pairs, either in-person or on the phone. The final interviews with Family A were at a participant’s home, and all other in-person interviews were at the first-author’s office.

Participant capacity was explored when obtaining consent before each interview and, in the days after interviews, concerns arising from responses to standardized questionnaires were sensitively discussed on the phone, reminding participants about independent support, and the option of withdrawal from the study. In three cases, when coping was discussed after the second interview in response to identified vulnerability, participants indicated that they wanted to continue participation. To assist, the dates of their final interviews were adjusted, with participants preferring to meet in the months after the anniversary.

## Findings

After the family narrative is disrupted by sudden death (Neimeyer et al., 2014), families refer to features of their lives before the crisis (Backstory), aspects related to their relative’s death (Story of the death), and emerging characteristics of individual and family life (Ongoing adjustment) to reconstruct their narrative (Walker et al., 2013). Codes and categories emerging during inductive coding were therefore sorted using this longitudinal framework.

A summary of coding outcomes related to Backstory and Story of the death is presented in Table IV, and those related to Ongoing adjustment are listed in Table V. For readers interested in further details, Supplementary File 2 contains concrete examples of family experiences at the hospital and during the months that followed. In Sections 1 to 3 below, we explore emergent categories using some concrete data. Readers preferring an abstract overview of family functioning and coding can bypass these detailed descriptions and refer to the *Consolidation of findings* before the **Discussion**

**Table IV.** Outcomes of coding related to the backstory and story of the death.

Longitudinal framework	Codes and categories emerging during inductive coding of interview transcripts	
	Categories	Codes
Backstory	The family as it was	Patient characteristics
		Family structure
		Relationships with the patient
		Knowledge about OD
Story of the Death	Critical incident and hospital context	Unfolding crisis
		Initial reactions
		Receiving the bad news
		Understanding the in-hospital context
	Relationships	Time with the patient
		Family togetherness
		Coordinating family and friends
		Connections with HCPs
	Potential for OD	Deciding about OD
		The last days of the patient’s life
		The time preceding WCRS
		WCRS
	After the patient’s death	Initial responses to the death
		Final farewell
		Leaving the hospital
		HCP grief
		Evaluating experiences

**Table V.** Outcomes of coding related to ongoing adjustment.

Longitudinal framework	Codes and categories emerging during inductive coding of interview transcripts	
	Categories	Codes
Ongoing adjustment	Continuing Bonds	Connection with the deceased
		Last conversations
		Fortunate occurrences
		Missed opportunities
		Sharing stories
		Decisions linked to their relative
	Individual grief experiences	Vulnerability
		Waves of grief
		Making sense
		Individual restoration orientation
	Interaction and relationships	Observing others
		Adjustments in relationships
		The value of openness
		Shared loss and restoration orientation
		Role of children
		Friendships
	Thoughts about IHPs, OD and recipients	Comforting and troubling thoughts
		Role of OD context in ongoing adjustment
		New relationships
		Recipients
	Stability and change	Monitoring grief
		Value of well-defined tasks
		Emergent growth

### Section 1: backstory

Section 1 introduces pre-existing characteristics of participating families, while Supplementary File 1 shares family genograms and brief descriptions of functioning before critical incidents. In addition to providing context for understanding in-hospital experiences and later family adjustment, these descriptions will enable readers to consider transferability of findings. Coding related to Backstory included one category, *The family as it was*, which contained the codes: Family structure, Patient characteristics, Relationships with the patient, and Knowledge about OD. Descriptions below consider the first three of these while Knowledge about OD is referred to in Section 2, because this played a role in family responses at the hospital. The significance of some other features of backstories became apparent during the year following the death. These included last conversations, fortunate occurrences, and missed opportunities, described in Section 3. Throughout the report, phrases used by participants are shown in italics.

#### The family as it was

Participants were all English-speaking Australians. Their relationship to the potential donor included siblings, spouses, parents, parents-in-law, and adult children. The patients included a 72-year-old male (Pierre) whose son participated, a 33-year-old female (Elsie) whose spouse and sister participated, a 22-year-old female (Mae) whose parents and siblings participated in the first interview with her mother then completing the second and third interviews after her father’s sudden death, a 17-year-old female (Alice) whose mother participated for the first interview before withdrawing from the study, and a 32-year old male (James) whose spouse, mother, brother, and parents-in-law participated for the full duration of the study.

Family life before the critical incidents was characterized by constructive interaction and secure relationships. All participants had future plans and enjoyed opportunities for personal and interpersonal growth. Readers wanting to know more about individual characteristics and family structure before critical events can refer to Supplementary File 1.

### Section 2: the story of the death

Section 2 explores critical events and time at the hospital where families heard about futility of treatment, considered OD, and attended WCRS. Coding identified four categories: Critical incident and hospital context, Relationships, Potential for OD, and After the patient’s death. We refer to these below to highlight factors that shaped each family’s story of the death.

#### Critical incident and hospital context

Critical incidents are described to facilitate understanding of the disruption of family narratives. Thereafter, exemplars demonstrate the remaining codes from Table IV. James and Alice experienced brain haemorrhages, Elsie and Mae were admitted following self-inflicted injuries, and Pierre suffered a head injury after a fall. In response to these crises, Helena, and Joanne, who were with James and Alice respectively, called for an ambulance, alerted relatives, and waited at the hospital. Elsie was receiving in-patient treatment for depression. Her doctor called Roland saying that she was missing, and he rushed to the hospital.

Having previously dealt with multiple crises, Pierre and Mae’s families found initial reactions difficult to calibrate. Steve heard late at night that Pierre had been admitted to hospital. Because there was often not much to do shortly after admission, he considered waiting until morning. When he received further news from the hospital, he got ready and left. Police let Stephen and Leanne know about Mae’s hospitalization. Anna recalls that, “When Mum and Dad woke me up, and said that Mae was in hospital, I asked them to keep me updated and went back to sleep.” When the situation became clearer, participants realized that their lives would never be the same. Anna said that once at the hospital, “I cried … it was only me, Mum and Dad. We knew it was bad even though the doctor had not spoken to us.” Family members who arrived after the patient was stabilized in ICU had difficulty understanding. Micah had travelled overnight and recalled, “I was a day behind. My parents and Anna accepted that Mae would die, but I struggled to make peace with that.”

#### Relationships

Participants treasured time with the patient and appreciated having a private room. Elizabeth recalled, “We sat with him listening to music and giving our loving farewells.” Family members watched each other and tried to find interaction that fostered togetherness. Paul said, “We responded to each other’s grief, sharing hugs and tears.” In some cases, a participant coordinated visiting times and kept others updated, while in other cases a family member who had some medical knowledge acted as a link between HCPs and the family. These roles were meaningful but also draining. Before WCRS Leanne, who had ensured that Mae was never alone, tactfully asked friends to allow close family to spend time with Mae.

**Table:** 

Stephen showed me a photo of the family holding Mae’s hand at the hospital. It had been Leanne’s idea to take the photo. Stephen was proud when he realized how it demonstrated the family’s solidarity. I appreciated the openness shown by participants.

In addition to focussing on the patient, ICU nurses provided support to families. Roland said that he appreciated the personalized nursing care, and recognized nurses by the different ways they performed their roles. Some participants spoke to social workers who were helpful. Where there were *glitches*, participants acknowledged that IHPs were complex for HCPs too. On the first night, Steve waited hours for Pierre to come out of surgery only to find later that Pierre was already in ICU. Steve said that he accepted that people make mistakes.

#### Potential for OD

Patients were intubated and transferred from the Emergency Department to ICU in attempts to save their lives. While they did not survive, this had significant implications, including preserving the potential for OD and providing time for family members, especially those travelling inter-state or internationally, to arrive. In some cases, family members raised OD. In others, after determining that treatment was futile, doctors communicated the prognosis to family members and then either mentioned OD or introduced the family to a DSNC. When considering OD, in addition to the potential to help others, participants focussed on the patient’s wishes, considering family knowledge and attitudes, and ensuring family harmony.

In some families, the family decision-makers had made many decisions together in the past and were able to rely on familiar decision-making patterns when considering OD. In other families, the patient had historically played a key role in decision-making, and the family faced the challenge of trialling new decision-making processes at the hospital.

James and Elsie’s families were assisted by registrations on the Australian Organ Donor Register. The families of Mae and Alice concluded that because they were caring, they would have supported OD. Pierre had not given directions regarding OD and his family found themselves in *uncharted territory*.

Canberra has no transplant centres. After family consent, DSNCs communicated with centres in other states to coordinate IHPs, arrange for surgeons to fly in, and later transport organs to transplant centres. Participants found that although processes were delayed, WCRS simultaneously seemed to rush towards them. They suggested that more advice on what to expect and how they could use their time before, during, and after WCRS would have helped. For example, delays near the end made planning difficult when Joanne tried to arrange a final good-bye in a loving environment. She was unsure when to ask Alice’s friends to leave because their presence was comforting, but the family needed time alone. While participants felt that DSNCs had prepared them for WCRS, the experience was stressful. Helena recalls, “They could not predict how long it would take James to pass away. To my horror, they said that if he took longer than 90 minutes, OD would not be possible. I dreaded the thought that James could struggle that long.”

#### After the patient’s death

Several participants recalled their relative’s last breath, with sadness because their life had ended, and relief because they were no longer suffering. Initial responses to the death were often hesitant and uncertain. After her death, Roland followed Elsie’s bed into the corridor leading to theatre and felt proud when he saw the row of HCPs forming an honour guard. Some participants decided not to see their relative after surgery, while others wanted to be there. Therese found the nurses respectful and caring when James returned from theatre. Being together at this final farewell was valuable to her, Helena, and Samuel. Steve remembers that when preparing to leave the hospital, “ … they offered us support, but we declined because we were okay and had received the support we needed.” Participants recalled instances of HCPs responding emotionally to the patient’s death. Anna said that “After Mae died, an OD nurse came in and thanked us. She was choked up.”

Participants generally were grateful for the care received and described their in-hospital experience as positive under the circumstances. In addition to interviews, the BPNA enabled participants to evaluate IHPs. Several reported that when with the patient, and when involved in patient care, they felt able to protect their relative, while when separated they felt powerless. Participants generally felt that HCPs worked together as a team. However, some noticed differences in information shared, or overheard staff discussing plans before sharing them with family. Some felt that the treating team could have been more available and used simpler language, and those who were not key decision-makers found that HCPs sometimes excluded them when introducing themselves and explaining matters. Those who approached staff found HCPs eager to help whereas those who waited for staff to involve them were sometimes disappointed. Other participants had not expected assistance with emotional support or anticipatory mourning, seeing these as family processes.

**Table:** 

After the first research interview, Roland indicated that exploring events preceding Elsie’s death and completing the BPNA had been stressful, but nevertheless meaningful. His comments confirmed the importance of reminding participants that they could take a break or choose not to answer questions that they found too sensitive.

### Section 3: ongoing adjustment

While Sections 1 and 2 reported on experiences occurring before meeting the researcher, Section 3 explores events unfolding while participating in the study. Codes related to Ongoing adjustment are listed in Table V where they are organized according to five emerging categories: Continuing Bonds; Individual grief experiences; Interaction and relationships; Thoughts about IHPs, OD and recipients; and Stability and change.

#### Continuing bonds

Psychological connection with their deceased relative was important to participants. Fond memories fostered positive closeness, but unexpected, intrusive memories were distressing. Elizabeth noted that, “My connection to James is strong with many memories of happy times together.” On the other hand, she also said that although she gradually made progress in containing her grief, some sudden memories or thoughts were overwhelming.

Several spontaneously described their last conversations with their relative. Some described pre-death events, interpreted as fortunate occurrences, as comforting. For example, months before Mae died, Anna and Mae’s relationship became tense, and the family was glad that they had reconciled before Mae’s sudden death. Conversely, events that were seen as missed opportunities caused distress. In the second half of the year following Pierre’s death, Steve experienced negative rumination and regret. He knew that he had done what he could while Pierre was alive, but intrusive thoughts and critical questioning *drowned out the logic*.

Sharing stories helped participants organize thoughts and make sense of their relative’s life and death. Paul prepared James’ eulogy: “I wanted to give my brother a good farewell and highlight happy memories and funny stories.” When making decisions, participants found themselves considering what their relative would have done. For example, Peggy said, “At times you ask yourself: What would she do in this situation?”

**Table:** 

Peggy experienced tearful moments during interviews but wanted to share her story. We used short pauses when necessary to make this easier. Roland asked whether policymakers would be told about the research. Participants’ commitment to the project motivated me to make their stories visible by publishing in an open access journal.

#### Individual grief experiences

Especially during the first few months, waves of grief were triggered unexpectedly contributing to vulnerability. Shortly after Alice’s death, Joanne found no predictable pattern in her grief. Some days at home were quiet and relaxing while others were emotional and difficult. Some used metaphors to make sense of personal change. After completing several post-death tasks, Steve said “The *dust is settling* … I am looking out over a *barren plain*. I should move forward and fill it with my life, but I am not sure how to.” Participants gradually found that activities such as returning to work and making present-oriented decisions contributed to direction, purpose, and hope. Peggy found it comforting to contribute to Hannah-Kate’s life and enjoyed telling her about Elsie.

#### Interaction and relationships

Family members observed each other and adjusted interaction to improve fit. Therese and Samuel felt relieved when their youngest son went to stay with Helena. She was not alone, and they could hear from him about her coping rather than bothering her. When family members were together, openness and shared restoration-oriented activities fostered togetherness and reduced the sense of overload. After Stephen’s death, Leanne motivated her children to help sort Mae’s belongings. “I was glad that we did it together. It brought many good memories, laughter, and tears. It was a good bonding session.”

Although family members under 18 years were excluded as participants, descriptions indicated that children’s vulnerability and spontaneity prompted openness from adults. Elizabeth’s grandchildren played an important role in her life. Looking after them not only gave her direction, but she enjoyed telling them about their Uncle James. Several participants valued being able to talk to friends. Peggy could not always speak freely to her parents as she did not want to upset them but could express herself when with friends.

**Table:** 

While Paul usually did not share his inner thoughts with others, he said that interviews provided a space where he could hear himself putting his story together. As Paul and I discussed the overlap between his bereavement and research experiences, I aimed to keep my roles as researcher and therapist separate and realized that this was not always easy.

#### Thoughts about IHPs, OD and recipients

Some wondered about implications of in-hospital events or how to provide feedback to treating teams, while others took cards to the ICU and arranged meetings with DSNCs. Several described troubling thoughts. Elizabeth, for example, spoke to doctors to clarify whether James’ stroke could have been avoided, and explanations that she received helped her find closure. Some participants struggled to recall details of IHPs and described time at the hospital as draining. Others remembered active participation and family-led rituals, which contributed to memories of family togetherness and a family-centred story of their relative’s death. Helena was comforted by the thought that James died peacefully and was not alone.

Where transplants occurred, participants said that helping recipients and their families was an important positive aspect to their sad experience. Some met other donor families and recipients at events like a walk to raise OD awareness, or a Service of Remembrance. Even when participants did not attend, these events acted as milestones prompting honouring of their relative and reaffirming hope for recipients’ health. Hearing about how transplantation changed lives reinforced Roland’s commitment to his decision. He said, “When Elsie died, I got accepted into a new family.” James’ family received an anonymous card from a recipient. This was comforting and Helena hoped to hear from others. When there were no further letters, she thought about writing to recipients, but was not sure what to say. She considered that recipients may experience similar hesitancy. Later in the year, the family asked for updates and were pleased to receive de-identified confirmation that recipients were well.

#### Stability and change

Participants monitored changing grief and sense of capacity. Peggy said, “Usually we have a big Christmas at Mum and Dad’s, but we did not feel ready for that. Instead, Mum and Dad came to us.” Having well-defined and manageable tasks helped them to observe progress, experience some stability, and increase capacity. After retiring, Elizabeth worked casually for a few weeks. The work was not demanding but nevertheless gave her a sense of direction. In several cases, personal and relational growth gradually emerged. Paul had previously been logical and task-oriented and later appreciated the value of emotional expression in his relationships with Elizabeth and Yvonne. Towards the end of the year, participants could make pro-active plans. Elizabeth and Anthony, for example, arranged a holiday together.

### Consolidation of findings

While findings have been presented in separate sections according to the longitudinal framework, the study highlighted how aspects of historical family life and in-hospital experiences played a role in ongoing individual and family adjustment. To demonstrate this, an overarching narrative illuminating family experiences across time is presented. This narrative presents findings in a more abstract rather than concrete way but nevertheless follows the order and structure of the codes and categories identified earlier. When appearing in the narrative, categories are written in italics for ease of reference. After the narrative, emerging themes that connect categories and codes are described.

### Overarching narrative

Backstories described *families as they were* before critical incidents. Participants had experienced confidence, secure relationships, and self-sufficient family units. Parents of young children found meaning in observing their children’s increasing capacity, while parents of adults worked at balancing being involved and respecting their children’s independence. Family members proactively avoided tension by considering their future together when making decisions.

Codes related to the Story of the death included *critical incidents and the hospital context*, where participants reported initial reactions such as symptoms of acute stress and being overwhelmed by the unfamiliar environment. Early responses to the unfolding crisis included hope for the patient’s survival, but realization that even if they survived, life would not be the same. Some were separated from the patient for what seemed to be long periods of time without receiving updates. Family members noticed that HCPs sometimes seemed hesitant to discuss matters such as the patient’s prognosis.

Although hearing about their relative’s imminent death was devastating, participants appreciated openness and clarity. To those arriving after the patient’s transfer to ICU, their relative appeared to be sleeping peacefully, which seemed incongruent with explanations about the futility of treatment. Ongoing and consistent information was vital and when family members observed the treating team working together, trust was strengthened. Where a family member acted as intermediary between the family and HCPs, staff support better fitted family needs. Family members ensured that the patient was never alone and offered each other emotional support. Apart from the immediate benefits of togetherness, *relationships* strengthened while families made shared sense of their experiences at the hospital.

When the *potential for OD* was explained, pre-existing family knowledge (especially about their relative’s preferences) and understanding of in-hospital processes influenced decision-making and other tasks. While participating families appreciated opportunities for togetherness before WCRS, the time was simultaneously draining. Although watching their relative die was difficult, some took the opportunity to shape the experience. Nevertheless, several participants described uncertainty about what to do immediately *after the patient’s death*.

Related to Ongoing adjustment during the months that followed, the development of post-death *continuing bonds* with their relative were highlighted by all participants. Thoughts about last conversations, and perceptions of fortunate occurrences or missed opportunities were either comforting or distressing. When family members got together, it was meaningful to share stories and honour their relative. Togetherness, active participation, shared decision-making, and interactive meaning-making at the hospital was seen to have reflected closeness to their relative contributing to a peaceful death and family solidarity. At the same time, ambiguities and incomplete understanding of in-hospital events contributed to ongoing uncertainty and anxiety until resolution was found.

Early weeks included instability as participants struggled to make sense of their loss and adapt to changed roles. *Individual grief experiences* included strong emotions that were triggered unexpectedly, and intrusive ruminating that was unhelpful but difficult to avoid. Over time, individuals experienced the gradual emergence of greater capacity for openness and completion of daily tasks.

*Interaction and relationships* gradually evolved. Participants observed each other and tried to find interaction that would be mutually satisfactory. When respect and openness was shown, participants felt validated. Shared activities contributed to meaning-making and facilitated positive relational change. Participants noted that some novel behaviours or interaction initiated at the hospital were later drawn into family life, influencing individual and relational growth.

Participants described spontaneous *thoughts about IHPs, OD, and recipients*. Where transplants had occurred, the possibility to have contributed to something positive amid their painful experience was valued. Some requested de-identified updates and found it meaningful to hear about recipient progress. Several participants exchanged anonymous letters with recipients or became involved in activities related to OD in general, making new connections.

Over the year, participants experienced both increasing *stability and periods of change*. They monitored their changing grief and identified factors that seemed to contribute to a greater sense of control, including well-defined tasks. A year after their relative’s death, several described features of personal growth, relational change, and family resilience compared to when grief was at its strongest. Nevertheless, all participants experienced vulnerability and sadness related to opportunities that their relative had missed out on.

### Emerging themes

Six minor themes connect the inductively coded categories across the *a priori* chosen time periods of Backstory, Story of the death, and Ongoing adjustment. These are depicted in Table VI, and include diversity across individual and family experiences, reconstructing coherent narratives, completing the deceased’s biography, developing and navigating a post-death relationship, the gradual increase in individual confidence and capacity to connect with others, and the co-existence of vulnerability and resilience as individual family members and the family system adjusted at the hospital and over the months following. In turn, three overarching major themes, (1) navigating grief, (2) hope for the future, and (3) meanings arising in the OD context, emerged. These are seen as connected to each other and running as threads through the minor themes.

**Table VI.** Emerging minor and major themes

Minor themes
Diversity across individual and family experiences Reconstructing coherent narratives Completing the deceased’s biography Developing and navigating a post-death relationship Gradual increase in individual confidence and capacity to connect with others Co-existence of vulnerability and resilience
Overarching major themes
Navigating grief Hope for the future Meanings arising in the OD context

## Discussion

In this section we elaborate on themes identified in the Findings section. We firstly introduce the major themes of *Navigating grief; Hope for the future*, and *Meanings arising in the OD context* by providing descriptions of (i) the fit between grief theory and family experiences, (ii) the evolving nature of hope, and (iii) the influence of the OD context. Further discussion of these themes is then spread across the discussions of the minor themes. Given our overall aim of identifying and highlighting opportunities for care, the discussion of Diversity at the hospital, which contributes directly to our Recommendations for practice later, is thorough and shares multiple leverage points. The minor themes related to family bereavement over time are each introduced separately and further demonstrated in Supplementary File 3 using concrete examples of participant experiences.

### The fit between grief theory and family experiences

Bereavement-related tasks identified by Worden (2018) were clearly demonstrated at the hospital, with participants initially struggling to come to terms with the prognosis of imminent death and experiencing the pain of grief. The development of a post-death bond (Silverman et al., 1996), the completion of the deceased’s biography (Walter, 1996) and the navigation of tasks in a world without their relative (Worden, 2018) began at the hospital too, laying the foundation for their further engagement in loss-oriented and restoration-oriented activities (Stroebe & Schut, 1999, 2015). At the hospital and thereafter, family members observed each other and monitored each other’s coping as they co-regulated emotions and made changes to their patterns of interaction (Barboza et al., 2021; Finch, 2007). The ongoing process of adjustment over the year following the death could be described with reference to meaning-making, systemic change, and grief theory. Participant descriptions highlighted ways that time at the hospital had provided opportunities to navigate grief together in a supportive environment, potentially providing a foundation for later experiences.

### The evolving nature of hope

At the hospital and over the year that follows, hope transforms in response to unfolding events (Jensen, 2016; Walker & Sque, 2016). Before the critical incident families had hopes and future plans, and then at the time of the critical incident they hoped that a quick medical response would save their relative. After gaining an understanding of the imminent death, participants hoped that their relative did not experience pain. When deciding about OD, family members hoped to make decisions that would fit with their relative and benefit others. Deciding together as a family reinforced hope for future togetherness and family resilience.

In response to grief, family members hoped to make mutually satisfactory adjustments in behavioural patterns. Where transplants had occurred, they hoped for the recipients’ health, and some hoped to receive correspondence from recipients. As they adjusted, participants hoped that individuals and the family system would be able to function well as time went by.

### The influence of the OD context

While it had been determined that patients would not survive, WCRS was delayed enabling their families to consider OD. This also meant that family members had time to arrive at the hospital, be together, and spend time with the patient. Several participants described using this time for activities of anticipatory mourning that were remembered fondly in the months thereafter. Consideration of OD would have been one of the families’ first shared decisions in a world without their relative. Guidance received was valued as family members shared ideas and stories in a respectful and supportive environment while making their decision. This fostered family solidarity, and a connection to their relative with all families wanting to decide in a way that would fit their relative’s wishes and characteristics. Being at the bedside when their relative died was difficult but meaningful and contributed to memories of a peaceful death. Several participants highlighted ways in which aspects of the interaction in the supportive hospital environment were drawn into family patterns during the year that followed. Most families mentioned the time together at the hospital and spoke about OD at the funeral and other events where their relative was honoured, demonstrating ways in which these features became part of the family narrative, the story of the death, and the deceased’s biography.

Similar to previous findings (Dicks et al, 2018a); Kentish-Barnes et al., 2019b, 2019c), where transplants had occurred, family members found it meaningful to hear about recipient progress, which confirmed that something good had come from their sad experience. While they did not meet the recipients of their relative’s organs, some participants exchanged anonymous correspondence with them. Some of these found that the writing process was quite complex confirming that, as suggested by Galasinski and Sque (2016), some assistance may be appreciated by those who wish to write.

Others attended events arranged by the OD agency where they met other donor families and transplant recipients. These events were meaningful, confirming the impact of decisions such as those made by the family, and contributing to acknowledgement and supportive relationships beyond the family’s pre-existing support system.

### The minor themes

Discussion of the minor themes has been organized under the headings of Diversity at the hospital, Unexpected death and the family narrative, The deceased’s biography and story of their death, Stability and change in the post-death relationship with the deceased, Posttraumatic growth, and Individual vulnerability and family resilience. We discuss these minor themes with reference to pre-existing OD literature and the study’s guiding frameworks while connecting the time periods to make sense of the longitudinal unfolding of family experiences. Readers will find reference to the major themes within these discussions. Concrete exemplars of minor themes are available in Supplementary File 3 for readers interested in exploring how the abstract discussions below can be related to family experiences.

### Diversity at the hospital

As described by the Australian Organ and Tissue Authority (Australian Organ and Tissue Authority (AOTA), 2021), treating teams and donation coordinators internationally refer to best practice guidelines when caring for the potential donor and providing compassionate support to their family. Nevertheless, while HCPs who supported participating families in this single-centre study would have received similar training and used similar policies to guide their actions, participants reported diversity in their experiences at the hospital. In addition to the role played by pre-existing factors such as knowing their relative’s donation preferences, participants highlighted the importance of in-hospital variables as role players observed each other (Finch, 2007), and co-regulated their behaviours, searching for interaction that would fit (Barboza et al., 2021).

When we explored the system that formed at the hospital, we identified several factors that contributed to diversity. These included the family’s understanding of the patient’s prognosis, HCPs’ perceptions of family coping, staff confidence and ability to manage complex roles, the degree of disruption to family decision-making structures, whether or not an intermediary connected staff and family systems, and the nature of family meaning-making in relation to OD and IHPs. These are discussed below:

As other researchers have shown (Lagacé et al., 2018; Mills & Koulouglioti, 2016), ongoing and consistent information was vital to orient families to the in-hospital context. As has previously been found (Ralph et al., 2014; Sque & Payne, 1996), to those who arrived after their relative’s transfer to ICU, the patient appeared to be sleeping peacefully. The importance of resolving ambiguities and gaining a clear understanding of the patient’s prognosis is highlighted by Worden’s (2018) first task of bereavement: Accepting the death.

In this regard, differences between DCDD and DNDD have previously been found to influence responses of family members and HCPs (Siminoff et al., 2017; Verble et al., 2020). As described by Pratt et al. (2019), some participants in the current study reported concern that further treatment would keep their relative alive but functionless. Others worried that, given that the patient was not dead, delays would prolong suffering. The emergence of these meanings supports observations that relatives of DCDD patients feel responsible for deciding about the patient’s life and death (Lind, 2019; Verble et al., 2020). These participants felt anxious and focussed on being with their relative while coming to terms with IHPs.

Another meaning made by some participants was that their relative was alive, but unable to survive. These families accepted that their relative’s condition would not improve, could not be prolonged indefinitely, and did not include suffering. Participants from these families experienced less anxiety and used more opportunities for anticipatory mourning and interaction with the patient.

Perceptions of family anxiety or confidence in turn influenced reactions from HCPs. This is congruent with Siminoff et al. (2017), who found that when family members appeared vulnerable, staff seemed hesitant to approach with suggestions. In addition, in the present study, participant descriptions suggest that when family members were able to rely on pre-existing patterns of interaction and decision-making they may have appeared confident, again contributing to staff taking a step back.

**Table:** 

During recruitment, several families were seen to be too vulnerable to be introduced to the study, confirming that perceptions of vulnerability influenced HCP-family interaction.

On the other hand, in cases where hospital staff seemed to evaluate the family as being vulnerable enough to need assistance, and resilient enough to engage in patient-care and other activities, HCPs acted more confidently. Suggestions were made and activities were adjusted to facilitate family involvement. This occurred more often in cases where a family member acted as an intermediary between the HCP and family systems. In these cases, family understanding was improved, HCP responses to family needs fitted better, and families were more aware of opportunities at the hospital.

The level of engagement with HCPs has been found to be affected by other factors too, including staff training and HCP confidence (Maloney & Altmaier, 2003; Simpkin et al., 2009). In addition, researchers have found that while patient care in the ICU is greatly appreciated, family members sometimes feel that family needs are overlooked (Fernandes et al., 2015; Ma et al., 2021; Ralph et al., 2014). This may occur because, if the family consents to OD, donation coordinators must attend to many tasks related to the donation and transplantation processes, and ICU nurses may feel that they have the responsibility to honour the family’s wish by maintaining medical aspects of the potential donor’s functioning. If family members decline OD, donation coordinators will withdraw, respecting the family’s decision. In contrast, previous researchers have argued that family care in preparation for death, before WCRS, while the patient is dying, and after their death is vital (Bloomer et al., 2017; Meeker, 2020; Prescott et al., 2019), and the value of a final farewell has been stressed (Forsberg et al., 2014).

Several participants in the present study, however, said that while HCPs had fostered clear understanding of OD processes, the family would have appreciated more advice and assistance regarding use of their time before, during, and after WCRS, highlighting the potential value of a support coordinator with a focus on bereavement care (Kelso et al., 2007; McAdam & Puntillo, 2018). This is significant because active participation (Noome et al., 2016) and identifying positives amid a tragedy can support meaning-making (Gillies & Neimeyer, 2006). In addition, care that fosters togetherness, respect, mutual support, and a sense of agency has a positive influence on ongoing adjustment (Cook et al., 2015; Hinkle et al., 2015; Lopez et al., 2018; N. Y. K. Yeo et al., 2021).

Participant descriptions suggest that families may find aspects of their time at the hospital comforting, regardless of their decision. These include opportunities for family togetherness, time with the patient, meaning-making opportunities, and facilitating a peaceful death. The longitudinal study illuminated the influence of these experiences on family adjustment in the months that followed. For example, while some remembered the IHPs as long and draining, other participants who had been actively involved at the hospital had better recall, especially of positive moments with the patient and other family members.

In some cases, while navigating in-hospital processes, family members had needed to develop new ways of interacting and making decisions before demonstrating to the extended family that their choices were suitable (Carroll & Burton, 2000; Coetzee & Van Niekerk, 2018; Finch, 2007). When interaction initiated at the hospital fitted with family values and assisted the family, it was later drawn into family life, influencing ongoing adjustment. We propose that, when experiencing both disruption in decision-making patterns and uncertainty about the patient’s OD wishes, families would benefit from additional assistance during decision-making (De Groot et al., 2016, 2015; Leal de Moraes et al., 2019, 2018). This is important because impulsive decisions, associated with anxiety and uncertainty, can contribute to significant stress later (Cleiren & van Zoelen, 2002; Kesselring et al., 2007).

As Walker et al. (2017) have noted, participants reported that efforts made by HCPs to acknowledge their OD decision and their relative’s potential to help others were appreciated. On the other hand, Taylor et al. (2018) have found that when OD via the DCDD pathway does not proceed for medical reasons, some consenting families struggle to make sense, while others may find comfort in tissue donation. In the one participating family where OD could not proceed, family members developed the shared view that time at the hospital had assured them that nothing would have saved their relative, contributing to peace of mind.

### Unexpected death and the family narrative

Individual and family narratives contribute to a sense of identity and belonging (Neimeyer et al., 2014). When sudden death disrupts these narratives, processes of meaning making and identity change are initiated in attempts to re-establish coherence (Park, 2010). Participants described meaning-making and narrative construction as ongoing tasks. At the hospital, for example, family members made sense of the need to decide about organ donation as something that could demonstrate family togetherness and respect for their relative’s characteristics or registered wishes. Over the months that followed, metaphors, dreams, and memories of time at the hospital were drawn into individual and family narratives.

### The deceased’s biography and the story of their death

Walter (1996) argued that the bereaved must complete the deceased’s biography. In this regard, participants highlighted how IHPs and family interaction at the hospital became part of the story of their relative’s life and death. At the funeral and when explaining their experiences to friends, participants highlighted how being able to spend time with the patient and ensure that they did not die alone had been important to the family. When transplants had occurred, the donor’s role in saving others was included as part of their biography, and links between this final act and the way they had led their lives were highlighted.

### Stability and change in the post-death relationship with the deceased

As has been described by previous researchers (Silverman et al., 1996; Stroebe & Schut, 2005), continuing bonds with the deceased were experienced as part of participants’ bereavement. At times when they felt regret over things that had not been said or done, the bond contributed to guilt and worry, while at times when participants recalled positive memories, the experience of connection was comforting. As noted by Mitima-Verloop et al. (2021), providing a eulogy or creating a remembrance garden facilitated welcomed closeness.

### Posttraumatic growth

As has been suggested by other researchers (Dyregrov & Dyregrov, 2019; Tedeschi & Calhoun, 2004; Walsh, 2002), participant adjustment included growing capacity and post-traumatic growth. Nevertheless, these processes were not easy, and included reorganization of values, identity, and relationships. Participants generally found themselves responding to what was immediately ahead of them, and only when looking back at the end of the year could they truly appreciate the changes that they had made.

**Table:** 

Leanne said to me, “It’s good to hear that what I’ve been through makes sense … when you’re living through it, it’s chaos in a way.” While I had viewed rephrasing participant experiences as a way of checking my understanding, Leanne’s comments highlighted the need for caution given that my summaries could alter participant perceptions.

**Figure 1.**
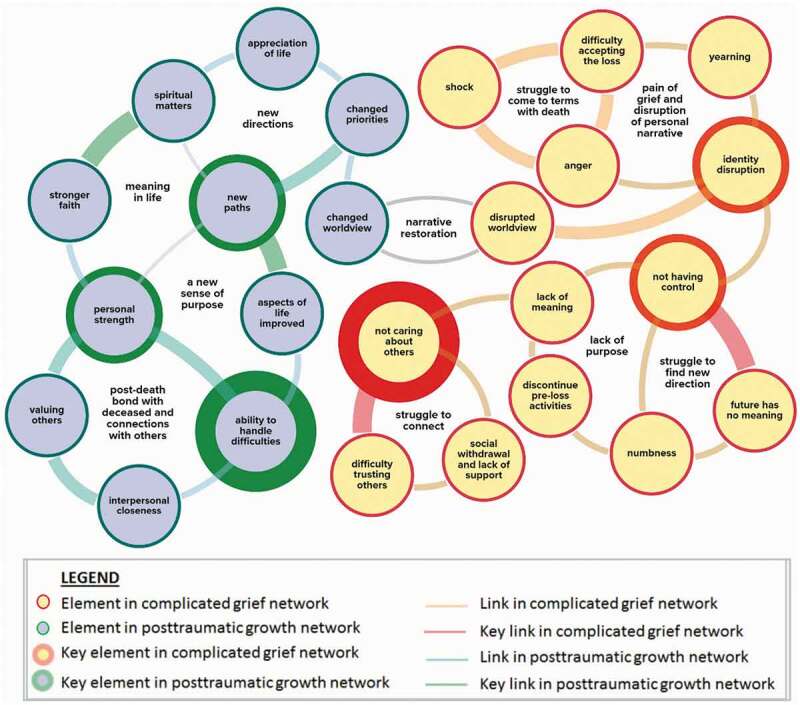
Relationships between posttraumatic growth and complicated grief. Adapted with permission from “Bereavement outcomes as causal systems: A network analysis of the co-occurrence of complicated grief and posttraumatic growth.” by B.W. Bellet et al. (2018), *Clinical Psychological Science, 6*(6), 805. (https://doi.org/10.1177/2167702618777454).

The idea that grief and growth co-evolve has been explored by Bellet et al. (2018), who performed a network analysis of the symptoms of complicated grief and signs of posttraumatic growth. They found that while sudden death disrupts one’s narrative, rebuilding one’s identity and worldview fosters personal growth. In Figure 1, those findings are applied to the adjustment described by participants in the present study. Elements and connections that Bellet et al. had identified as most significant are depicted as thicker circles and lines, respectively. Initial experiences included struggling to connect with others, yearning, identity disruption, loss of control and difficulty finding new direction. Over the months that followed, each in their own way, by monitoring their capacity, making use of available support, and carefully considering decisions, participants changed priorities, found new connections, and built a sense of personal strength.

### Individual vulnerability and family resilience

In addition to individual impacts, sudden death changes family structure, influencing roles and responsibilities (Berger & Weiss, 2009; Mendenhall & Berge, 2010). Adaptation requires the family to reorganize, meeting the needs of individuals, while simultaneously encouraging participation which fosters family cohesion (Figley & Figley, 2009; Hooghe et al., 2012; Papero, 2017). Rather than being able to follow usual patterns at the hospital and thereafter, families experienced a state of flux (Bonanno, 2004; Mark, 2011). This suggests that ongoing assessment of family strengths and vulnerabilities could enable fitting responses to changing family needs (Aoun, 2020; Aoun et al., 2015; Aoun et al., 2017; Poppe et al., 2019; Potter, 2018).

Researchers have found that when families encounter their first challenges without their relative at the hospital, a mediator can enhance relationships between family and staff, improving family confidence and facilitating a fit between staff actions and family needs (Dodd‐McCue, 2010; Dodd-McCue & Tartaglia, 2005; De Groot & van Hoek, 2017). This fit can contribute to a supportive environment promoting constructive individual adjustment and interactional change in the family system as family members make sense of their changed lives (Grisogono, 2006; Mehta et al., 2009).

Participants demonstrated that while each of the individual members of a family experienced vulnerability and attended to personal struggles, by adapting their interaction to fit each other’s needs, a sense of shared resilience emerged (Walsh, 2002). Although previous research has highlighted ways in which aftercare providers linked to the hospital or OD agency could assist individuals and families who seek help in the months that follow (Reed et al., 2015; Wind et al., 2022; N. Yeo et al., 2019), reports from families in the present study and earlier studies suggest that many family members may choose to find their own sources of support (Aoun et al., 2012; Frivold et al., 2016). This highlights the need to reinforce community resources in addition to improving support provided to families by donation agencies.

## Recommendations for practice

In the Discussion above, we connected emerging themes to literature, demonstrating how the chosen frameworks and literature from the OD field foster understanding of in-hospital experiences and evolving family adjustment. In relation to our research questions and working hypotheses, we feel that the identified themes illuminate key features of the bereavement of families of potential organ donors and support our working hypotheses. Referring to literature on trauma, and using the guiding frameworks chosen for the study, we identified several links between pre-existing family characteristics, the IHPs, and ongoing adjustment. Diversity at the hospital was influenced by factors such as the family’s understanding of the patient’s prognosis, HCPs’ perceptions of family coping, staff confidence and ability to manage complex roles, the degree of disruption to decision-making structures, whether or not an intermediary connected staff and family systems, and family meaning-making. These features can act as leverage points to facilitate opportunities for care that can have a lasting positive effect. Several examples are provided below:

Pre-existing family values, knowledge, structure, and dynamics influenced understanding, behaviour, and interaction at the hospital. We therefore feel that if HCPs were to get to know family characteristics early, they will be better positioned to shape the environment in ways that fitted with the family. Training should include enhancing psychosocial skills of HCPs and fostering an appreciation of systems thinking. Similarly, when family members arrive at the hospital following a traumatic event, an early trauma-informed response can enable them to understand the impact of trauma on individual and family functioning and find responses that reduce distress, stabilize the situation, and promote safety, connectedness, and agency (Petrinec & Daly, 2016; Wade et al., 2013).

The importance of the family’s time at the hospital was demonstrated from the perspective of grief theory too. We showed that the family’s grieving begins at the hospital where they can be assisted to accept the death of their relative, explore ways of managing the pain of grief (loss orientation), respond to tasks necessitated by the death (restoration orientation), and initiate the development of a post-death psychological bond with their relative. Systems theory highlighted the way that family members observe each other and co-regulate emotional and behavioural responses while searching for interaction that fits. The longitudinal nature of the study has shown that useful interaction and decision-making at the hospital that was found to fit with family values was drawn into interactional patterns later.

Therefore, in addition to helping families to make informed decisions about OD, the unique circumstances at the hospital provide opportunities to tailor support to the needs of the family as an adaptive system experiencing trauma, grief, unexpected change, and disruption of worldviews in an unfamiliar environment. Training that includes the frameworks introduced by our study can equip staff to identify and respond to a range of family needs that may otherwise go unnoticed. Given that ICU staff and DSNCs have many tasks to attend to, the importance of an intermediary who can focus on family needs and link the family and staff systems was highlighted. While in some families, this role may naturally emerge, other families would require a support coordinator from the hospital.

During the first year of their bereavement, several participants made use of activities such as awareness walks and Services of Remembrance offered by the OD agency. Some had questions about causes of the critical event or their relative’s death and several wondered about the outcomes of transplants and recipients’ ongoing well-being. It is important that families are aware of where to find answers to such questions, and assistance may be required for tasks such as writing anonymous letters to recipients.

In response to their grief, several participants were supported by their General Practitioners, work counsellors, psychologists, and friends. Because participants described the unexpected complexity of the IHPs, and because they sought help from providers outside the OD context, we feel that public education programmes and continuing professional development for services providers in the community should incorporate more information about experiences in the OD context.

## Study limitations and strengths

The study addresses an important gap by connecting various theoretical frameworks to the OD context. Inclusion of stakeholder input during study design, and involvement of participants during analysis and reporting stages increased the relevance and trustworthiness of the study. Detailed concrete descriptions and quotations from interviews that are included in Supplementary Files enable readers to consider the transferability of findings to other contexts.

However, only five families participated in this single-centre study. These did not include families where early investigation excluded the possibility of OD, those who had considered DNDD or declined OD. In addition, DSNCs did not provide PIFs when family grief or intra-familial stressors were significant. When commenting on the time before the critical incident, participating families described secure relationships. This has implications for transferability of findings too, considering that pre-existing functioning in other families may be less stable. We did not have the opportunity to learn directly from those families, or families who felt dissatisfied by IHPs. Nevertheless, exploring the adjustment of families who appeared to cope with in-hospital demands identified family and staff characteristics and actions that influence experiences, highlighting ways to improve family care in general.

Although no families who declined OD participated in the study, researchers have found that declining and consenting families experience similar struggles at the hospital (such as hoping for a peaceful death and family togetherness), suggesting that the introduction of grief, meaning-making, and systems theory would be useful lenses whether a family was to consent to or decline OD (Darnell et al., 2020; Leal de Moraes & Massarollo, 2009).

In only two of the participating families did more than one family member complete all stages of the study, hindering ability to observe system level coping directly. Nevertheless, the descriptions of participants provided information about their interaction with others in the family, enabling triangulation and trustworthy comments to be made about family functioning.

## Conclusions

While it is the potential for OD that initiates the unique in-hospital environment, that context contains features that may assist families with their bereavement in ways that are only indirectly related to OD. Training that includes the lenses of trauma, grief theory, systems theory, meaning-making and narrative will enable HCPs and families to identify and use opportunities for symptom reduction, active participation, anticipatory mourning, and meaning-making. This will allow family members to trial new behaviours and interaction in a supportive environment, while creating memories of family togetherness. Post-death bonds with the deceased will be initiated, and as they interact with each other, family members will learn about being a family in a world without their relative, laying the foundations for family adjustment. HCPs should balance being proactive and providing guidance with being in the background, so that families can shape the in-hospital environment in ways that contribute to a sense of family ownership.

While support from the OD agency was appreciated in the months that followed the death, several family members also sought assistance from sources outside the OD context. It is also noted that aftercare resources for families who have declined OD are limited. This highlights the need to provide education to service providers in the community to improve their understanding of the experiences of families of potential organ donors.

## Supplementary Material

Supplemental Material

## Abbreviations

AOTA: Australian Organ and Tissue Authority
BPNA: The Bereaved Parent Needs Assessment
DCDD: Donation after circulatory determination of death
DNDD: Donation after neurological determination of death
DPMP: Donors per million population
DSNC: Donation Specialist Nursing Coordinator
HCPs: Healthcare professionals
HREC: Human Research Ethics Committee
ICU: Intensive care unit
IHPs: In-hospital processes
OD: organ donation
PIF: Participant Information Form
WCRS: Withdrawal of cardio-respiratory support
