# Supplementary material for: The bereavement experiences of families of potential organ donors: a qualitative longitudinal case study illuminating opportunities for family care
Source: Int J Qual Stud Health Well-being. 2022 Dec 5;18(1):2149100. doi: 10.1080/17482631.2022.2149100 (PMC9731585; doi:10.1080/17482631.2022.2149100)
Supplement: Supplemental Material [file ZQHW_A_2149100_SM1322.docx]

**Supplementary File 1 – Pre-event family functioning**

**Backstories - *The family as it was***

Figures 1 to 5 show families before the critical incidents, with participants in yellow and patients in blue. Genograms are simplified with relatives who are later referred to by role (e.g., aunt) depicted by squares (males) and circles (females). Elsewhere, pseudonyms have been used.

| **Figure 1** |
| --- |
| *Family A* |
| 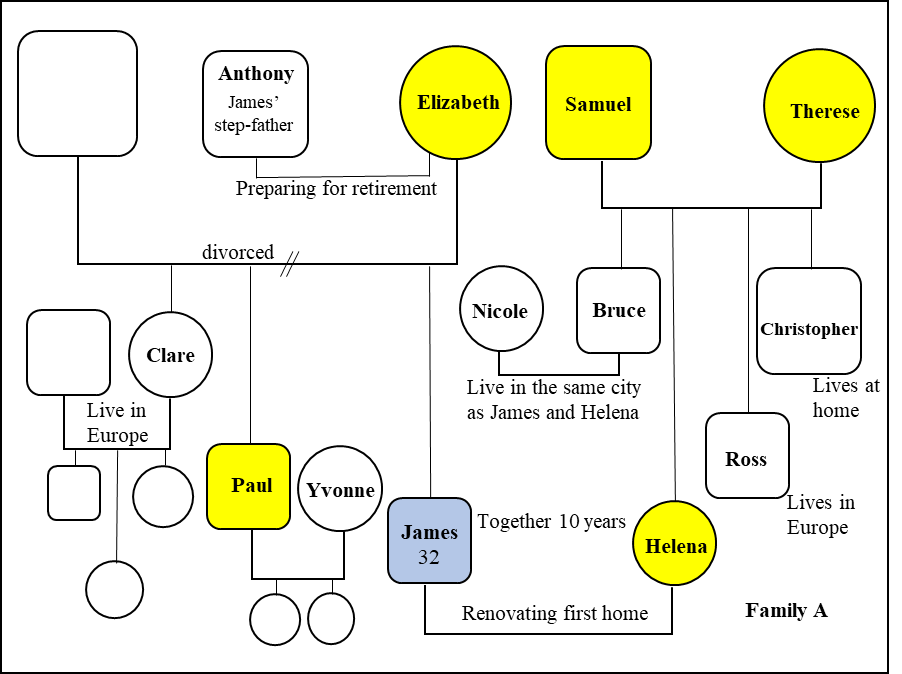 |

**Family A.** James (32 years) was intelligent, gentle, and kind. He and Helena had been married for two of their ten years together. They used a collaborative decision-making style and were excited about renovating their house, 600km from their hometown. Growing up, his older brother, Paul, had assisted James when he became anxious. James’ mother, Elizabeth, was preparing for retirement, and Therese and Samuel, Helena’s parents, were adapting to changing relationships with their four adult children.

| **Figure 2** |
| --- |
| *Family B* |
| 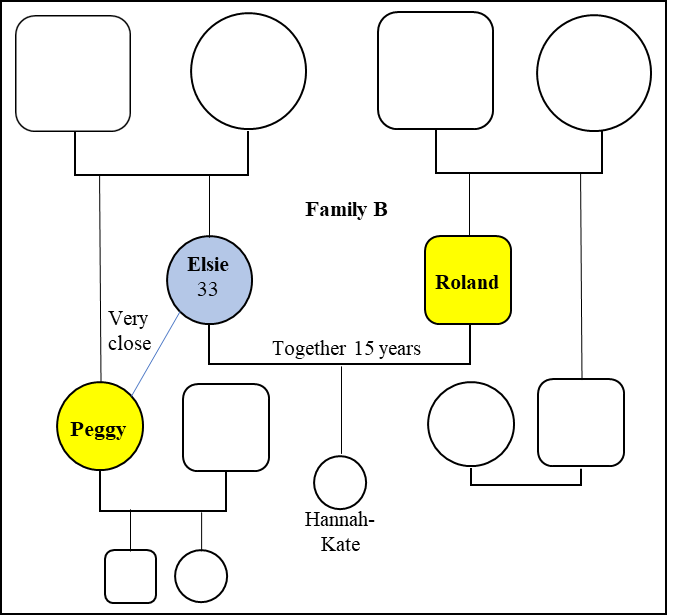 |

**Family B.** Elsie (33 years) enjoyed a strong relationship with her sister, Peggy. Elsie and Roland had been together for 15 years and were excited about being parents to Hannah-Kate. Elsie had been diagnosed with Bipolar Mood Disorder as a teenager, and sometimes had suicidal thoughts. Roland become anxious whenever she was depressed and then either his mother or Elsie’s mother stayed with them to provide support.

| **Figure 3** |
| --- |
| *Family C* |
| 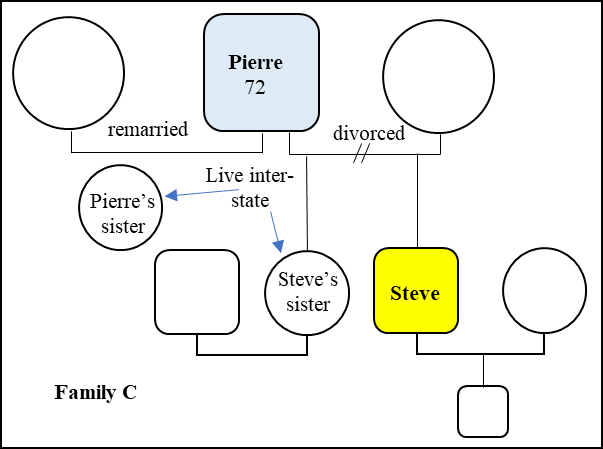 |

**Family C.** As he got older, Pierre (72 years) had several falls and health crises. Each time Pierre was admitted to hospital, his son, Steve, worked with his sister and their aunt, making careful decisions, and avoiding tension in the wider family. Steve had noticed that Pierre emerged from hospitalisations with *diminished returns*.

| **Figure 4** |
| --- |
| *Family D* |
| 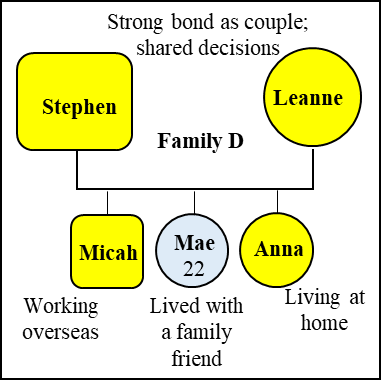 |

**Family D.** Mae (22 years) was bright, witty, kind, and creative. Anna and Mae were close, and as children sometimes listened to music with one earphone in Anna’s ear and one in Mae’s. Micah, who played a guiding role in his younger sister’s life, had a secure bond with her too. Stephen and Leanne had a strong relationship, enjoyed time with their children, and had meaningful friendships. Whenever Mae experienced strong mood fluctuations, the family worked together with help from extended family and friends.

**Family E.** Alice (17 years) attended school and had many friends. She enjoyed time with her parents and had good relationships with her extended family. Joanne and Garth were proud of their daughter who respected and appreciated their roles in her life.

| **Figure 5** |
| --- |
| *Family E* |
| 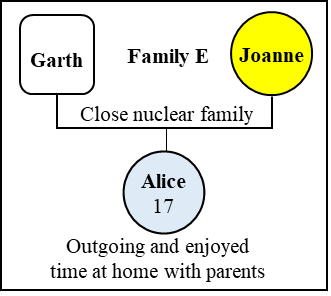 |

**Supplementary File 2 – Qualitative Coding**

| **Supplementary Table 1** | | |
| --- | --- | --- |
| *Coding related to Story of the Death* | | |
| CATEGORY | CODE | EXEMPLAR |
| Critical incident  and hospital context | Unfolding crisis | Described in Section 2 |
|  | Initial reactions | Described in Section 2 |
|  | Receiving the bad news | Helena remembers, “Our family had not arrived yet. … My friend, Kath, sat with me while the emergency doctor explained that James’ injury was *incompatible with life*.” Helena held onto hope until the ICU doctor confirmed the diagnosis *with tears in his eyes*.  Joanne found that HCPs were cautious: “No-one was prepared to tell us how bad things were. I called a friend’s father who is a surgeon, and he explained the seriousness. When I told family and friends, some kept telling me to have hope. |
|  |  |  |
|  | Understanding the in-hospital context | After Elsie’s admission, Roland found himself feeling powerless with the understanding that Elsie was almost dead. However, after observing nurses treating Elsie, it occurred to him that although she would not survive, she was still alive, although in an unfamiliar state.  After observing the way nurses were caring for Elsie, Roland viewed interaction with Elsie as meaningful, and was grateful that he, Hannah-Kate, and Elsie’s extended family would have a chance to be with her and later say good-bye. |
| CATEGORY | CODE | EXEMPLAR |
| Relationships | Time with the patient | Elizabeth recalled, “We sat with him listening to music and giving our loving farewells.” Paul accepted that he could not protect James as he had done before, and spoke in the presence of their family saying, “James, I love you. I am sorry that I can’t help you now.” |
|  | Family togetherness | Paul recalled: “We responded to each other’s grief, sharing hugs and tears.”  Joanne found it comforting to be with Alice at night when there were no other visitors.  Anna stayed with Mae the first night listening to music with one earphone in Mae’s ear, and one in her own. |
|  | Coordinating family and friends | Leanne coordinated visits from family and friends and reported that although it was draining, it felt right. Before WCRS, Leanne tactfully asked friends to wrap up their visits, allowing close family to be alone with Mae. Micah noted that he did not notify his friends. He said, “I needed to deal with what was happening without friends checking in.” |
|  | Connections with HCPs | Roland recognised nurses by the different ways they performed their roles, and noted, “… the nurse who was there the first day was on shift again on the third day. It was welcoming to see her again. She carefully washed Elsie, and then invited me and my mother to help.” |
| CATEGORY | CODE | EXEMPLAR |
| Potential for OD | Deciding about OD | After accepting the seriousness of Alice’s condition, Joanne asked about OD. This caught HCPs off-guard, and they appeared shocked.  Mae’s family considered her caring nature. Anna explained that “Mum and Dad were adamant that if we weren't *on the same page,* we wouldn't go ahead.”  Pierre’s family were unaware of his thoughts about OD, and viewed the OD request as *uncharted territory*, Steve recalls, “We didn't know how to answer that question, and we thought: What do we consider?” Steve described his family’s decision-making style as moving forward in *lockstep*. They had done this in response to previous hospitalisations and so, while deciding about OD was uncharted territory, the process used was familiar. |
|  | The last days of the patient’s life | Anna recalls being unprepared for the medical-social questionnaire: “All four of us were in the interview. Although we knew it was important, no-one realised how many questions there would be. I left early because some of the questions were too personal.”  During the last two days of Pierre’s life, his family had accepted that he was no longer present. They thought, “We don't see him … We see a body being kept alive.” |
|  | The time preceding WCRS | Helena and Elizabeth were committed to facilitating James’ OD decision. Nevertheless, Elizabeth found it confronting when imagining *all-in donation*. Because James wanted that and because she hoped something good would come from their painful experience, she made peace with the idea.  Joanne tried to arrange for the family to say good-bye in a loving environment. However, delays near the end made planning difficult. Joanne was unsure when to ask Alice’s friends to leave because their presence was comforting, but the family needed time alone. |
|  | WCRS | Helena recalls, “They could not predict how long it would take James to pass away. To my horror, they said that if he took longer than 90 minutes, OD would not be possible. I dreaded the thought that James could struggle for that long.” |
| CATEGORY | CODE | EXEMPLAR |
| After the patient’s death | Initial responses to the death | Roland had decided that he would leave the hospital after Elsie’s death. However, when she died, he was unsure what to do. He followed her into the corridor leading to the theatre and felt proud when he saw the row of HCPs forming an honour guard. |
|  | Final farewell | Therese found the nurses respectful and caring when James returned from theatre. Being together at this final farewell was valuable to her, Helena, and Samuel. |
|  | Leaving the hospital | Steve remembers that when preparing to leave the hospital, “… they offered us support, but we declined because we were okay and felt that we had received the support we needed.”  Roland remembered that as his family was preparing to leave, the DSNCs approached them and gave them badges acknowledging their decision to consent to OD. |
|  |  |  |
|  | HCP grief | Participants recalled instances of HCPs responding emotionally to the death of the patient. Roland said, “Staff were involved in the bereavement process because they developed a connection with Elsie before she died, and because their actions influenced her death and the family’s experiences.”  Anna remembered that “After Mae died, one of the OD nurses came in and thanked us. She was *choked up*.” |
|  |  |  |
|  | Evaluating experiences | Steve reported that staff were supportive, and he saw treatment as optimal. When there was a *glitch* on the first night, where he waited hours for Pierre to come out of surgery only to find that he was already in ICU, Steve accepted that people make mistakes.  In Pierre’s case, OD did not proceed for medical reasons discovered during surgery. Steve experienced some disappointment at first but reports that he and family members accepted that everyone had done their best. He and his sister were later relieved when they reframed delays as giving Pierre time to recover and leaving no unanswered questions. |

| **Supplementary Table 2** | | |
| --- | --- | --- |
| Experiences during the first year of bereavement – Ongoing adjustment | | |
| CATEGORY | CODE | EXEMPLAR |
| Continuing Bonds | Connection with the deceased | Elizabeth shared, “My connection to James is strong with many memories of happy times together. During the first months after his death, it felt like he could just walk in the door.” |
|  | Last conversations | Roland clearly remembered his last conversations with Elsie. Two nights prior to her suicide attempt, she had given Hannah-Kate a long bath as opposed to her usual task-oriented bath, and Roland had felt things were improving. |
|  | Fortunate occurrences | At some point, Anna and Mae’s relationship had become tense, and the family was glad that they reconciled before Mae’s death. |
|  | Missed opportunities | In the second half of the year following Pierre’s death, Steve experienced negative rumination and regret. This was confusing because on a logical level he knew that he had done what he could. However, intrusive thoughts, emotions and critical questioning drowned out the logic. |
|  | Sharing stories | Paul prepared James’ eulogy: “I wanted to give my brother a good farewell and highlight happy memories and funny stories. James cared deeply about others, and it was fitting that his final act helped six people.” |
|  | Decisions linked to their relative | Peggy described how decisions continued to be linked to Elsie: “You ask yourself ‘What would she do in this situation?’ Sometimes I talk to her … although I'm not getting a response, it's the some of the best times I have … It lets me feel close … “ |
| CATEGORY | CODE | EXEMPLAR |
| Individual grief experiences | Vulnerability | In the weeks following Alice’s death, Joanne struggled to identify a predictable pattern in her grief. Some days at home were quiet and relaxing while others were emotional and difficult. |
|  | Waves of grief | Elizabeth found that although she gradually made some progress in containing her grief, sudden unexpected memories or thoughts could take her back a few steps. |
|  | Making sense | After completing several post-death tasks, Steve said “The *dust is settling* … I am looking out over a *barren plain*. I should move forward and fill it with my life, but I am not sure how to.”  A year after Elsie’s death, Roland commented, “I wonder if I dealt with the first year as if it was *a work trip* … not allowing myself to miss her too much … just getting things done.” |
|  | Individual restoration orientation | Peggy found it comforting to contribute to Hannah-Kate’s life. She enjoyed telling her about Elsie and was glad that Roland left Hannah-Kate with her grandparents when he travelled. |
| Interaction and relationships | Observing others | Therese and Samuel felt relieved when their youngest son went to stay with Helena. She was not alone, and they could hear from him about her coping rather than bothering her. |
|  | Adjustments in relationships | Steve, who had previously not been an emotional person, said, “I talk to my partner. She's been with me when I've been sobbing away … she gives me new ideas.” |
|  | The value of openness | While Peggy was careful when she spoke to her parents, not wanting to upset them, with her friends there was common ground without intense emotions, and she could speak freely. |
|  | Shared loss and restoration orientation | After Stephen’s death, Leanne motivated her children to help her sort through Mae’s belongings. “I was glad that we did it together because the task brought back many good memories. There was laughter and tears, and it was a good bonding session.” |
|  | Role of children | Elizabeth’s grandchildren played an important role in her life. When she looked after them, not only did this give her direction, but she enjoyed telling them about their Uncle James. |
|  | Friendships | Helena found it meaningful to speak to friends who had known James and shared her sense of loss and confusion. |
| CATEGORY | CODE | EXEMPLAR |
| Thoughts about IHPs, OD and recipients | Comforting and troubling thoughts | Elizabeth and Helena agreed that “We miss James and are proud that he wanted to be an organ donor. It is comforting that our sad loss gave others a chance.”  Elizabeth spoke to doctors to clarify whether James’ stroke could have been avoided, and the explanations that she received helped her find closure. |
|  | Role of OD context in ongoing adjustment | After Stephen’s death, he and Mae were honoured at a local event and brief biographies were shared with the public, including the fact that they became organ donors and thereby helped others. This was meaningful to Leanne and Anna. |
|  |  |  |
|  | New relationships | Hearing about the ways that transplantation changed the lives of others reinforced Roland’s commitment to his decision. He said, “When Elsie died, I got accepted into a new family.”  On national “Thank You Day”, Peggy found that “It was beneficial to share Elsie’s story on social media, and it was comforting when people wrote back to say that they had registered their preferences on the national register.” |
|  | Recipients | In the weeks following Mae’s death, Stephen and Anna looked for something positive to come from their experience. They wrote an anonymous letter to recipients, and this was meaningful. |
| Stability and change | Monitoring grief | Peggy said, “Usually we have a big Christmas at Mum and Dad's, but we did not feel ready for that. Instead, Mum and Dad came to us.” |
|  | Value of well-defined tasks | After retiring, Elizabeth worked casually for a few weeks. She appreciated that the work was not demanding but nevertheless gave her a sense of direction. |
|  | Emergent growth | Towards the end of the year, Samuel and Therese reported that they appreciated life more, and had been spending more time with their adult children. Therese had been accustomed to planning ahead and shaping the future but came to appreciate the benefit of choosing actions based on their present value. Paul, similarly, had previously been logical and task-oriented and later appreciated the value of emotional expression in his relationships with Elizabeth and Yvonne. |

# Supplementary File 3 Emerging themes

The present file shares concrete exemplars of the minor themes, Reconstructing coherent narratives, Completing the deceased’s biography, Developing and navigating a post-death relationship, Gradual increase in individual confidence and capacity to connect with others, and Co-existence of vulnerability and resilience.

Exemplars are drawn from the narratives of the four families who participated for the full year following their relative’s death and together demonstrate Diversity across individual and family experiences. Aspects of the major themes, Navigating grief, Hope for the future, and Meanings arising in the OD context can be identified within the exemplars.

**Unexpected death and the family narrative**

Helena and James hoped to spend many happy years together. Elizabeth was weeks away from retiring and was excited to see the home renovations that James had told her about. Therese and Samuel wondered about what they would do with their independence when their youngest son moved out of the family home. Paul regularly spent time with his mother and looked forward to the soccer season when he and James would have lots to talk about. These narratives were disrupted by James’ sudden injury.

In the unfamiliar hospital environment family members initially felt overwhelmed. Paul’s wife, Yvonne, negotiated with doctors and got them to repeat their explanations when family members were confused. In time, participants developed a confident understanding and accepted James’s imminent death.

James had registered his donation preferences, and after accepting that he would die, Helena and Elizabeth hoped to be able to facilitate those wishes. Participants found staff to be caring and willing to accommodate family suggestions. Family members were pleased when they were able to spend time with James, showing respect and openness, contributing to family togetherness and a peaceful death.

In the weeks following James’s death, individual stability and family functioning were impacted and family members experienced intrusive thoughts and images while struggling to make sense of their lives. Family members hoped that they would individually and together cope better in the future. They also hoped that their research input would help other grieving families.

The family gradually connected emerging experiences to their disrupted narrative, and they adapted behavioural patterns in response to their changed lives. Pre-existing factors and interaction at the hospital seemed to provide a foundation for patterns that emerged. For example, Helena continued with the renovations, where following plans made with James kept her close to him. After working together at the hospital, Helena and Elizabeth remained close and found that their grief was similar.

When the family met at the anniversary of James’s death, shared meaning-making was appreciated. The family narrative included reference to how they had stood together at the hospital and supported each other in the time that followed. They were pleased that their donation decision had helped others and felt that this provided a positive amid their pain.

**The deceased’s biography and the story of their death**

At Elsie’s funeral, Roland and Peggy spoke about her experiences, including her battle with depression. While not denying the cause of her death, they focussed on her kind, hard-working and creative nature. Roland thanked the HCPs who helped her manage depression and expressed appreciation to doctors who had ensured that she survived long enough to get to ICU. He acknowledged the respectful care shown in ICU and expressed gratitude for the opportunity to shape the last days of her life. He highlighted the togetherness at the hospital where he had felt supported, and Elsie did not die alone. Roland and Peggy told funeral attendees that Elsie had registered her preferences on the OD register, and Roland said that he felt honoured to have been able to help her fulfil her wish to help others. In these ways, pre-existing individual and family characteristics, experiences in the supportive environment at the hospital, and features of Elsie’s death were drawn together in a coherent way to complete her biography. Later in the year, Peggy wrote a letter to recipients in which she shared anonymous information about Elsie, assisting recipients to understand the person who had helped them.

**Stability and change in the post-death relationship with the deceased**

At the hospital, Pierre’s family carefully considered his attitudes and hoped that he would have been satisfied with choices made. In the months following, Steve progressed through post-death tasks with a sense of achievement and felt connected to Pierre. However, when tasks neared completion, he became anxious. When repetitive negative thoughts and self-criticism contributed to regret and low mood, Steve found that spending time where his father’s ashes are scattered, and consuming alcohol facilitated closeness with Pierre. Later he accepted that this was unsustainable and adjusted his focus, determined to be a good father to his son. Steve’s account mirrors other participants’ experiences of comfort and struggles with intrusive thoughts and fluctuations as they navigated their evolving psychological relationship with their relative.

**Posttraumatic growth**

After Mae’s death, Leanne and Stephen depended on each other. They went the movies, visited friends, and spent time with Anna and Micah. However, when Stephen died a few months later, Leanne’s roles and routine changed. She explained, “Friends tried to support me. When I met with them individually this helped, but when their partners were present, it felt awkward.” Leanne later found it valuable to meet people who could get to know her as the person she was becoming.

She decided to sell the house and get a smaller place for her and Anna. Through her religion and friendships, she was part of a supportive community. Nearing the anniversary of Stephen’s death, she felt more resilient and accepted that the uncertainty and loneliness she sometimes experienced was part of her grief. Leanne’s experience shows openness and acceptance that while life was difficult, she had some control. Her perseverance through difficult periods contributed to decisions that fitted with her and Anna, and thereby to personal and relational growth.

**Individual vulnerability and family resilience**

Historically, Peggy and Roland’s interaction was shaped by their respective relationships with Elsie. At the hospital, Peggy understood technical aspects of the context, and this was comforting to Roland who was unfamiliar with the hospital environment. Peggy appreciated Roland negotiating with HCPs to arrange activities of anticipatory mourning for the family while she gathered and translated medical information. They collaborated and extended family members were satisfied with their decisions. These roles and the respectful collaboration shown shaped the family’s experiences at the hospital, and influenced interaction thereafter.

When Elsie died, Roland became a single parent to Hannah-Kate. In the months that followed, he and Peggy described a shared goal of helping Hannah-Kate to know Elsie. They regularly updated each other on personal progress and developments in the family. When he had to travel for work, Roland felt comfortable leaving Hannah-Kate with her maternal grandparents. Peggy described how important this was because “…not only did they lose their daughter, but they didn't know how often they would see Hannah-Kate.” Over time, these patterns were reinforced, and Peggy noted that, “… Roland lets Mum and Dad have Hannah-Kate for a few days every month … This is meaningful for them.” Roland said, “I need to balance being Hannah-Kate’s father and attending to other responsibilities because I was doing too much and becoming overwhelmed. I learnt to appreciate breaks from Hannah-Kate without feeling guilty.”

Roland reported that he saw a psychologist in the months following Elsie’s death, while Peggy found support from family and friends to be sufficient. Their experiences demonstrate the relationship between individual vulnerability and the emergence of family resilience when the needs of multiple family members are taken into consideration, and interaction is tailored to fit.
